# The Resurgence of Introgression Breeding, as Exemplified in Wheat Improvement

**DOI:** 10.3389/fpls.2020.00252

**Published:** 2020-03-06

**Authors:** Ming Hao, Lianquan Zhang, Shunzong Ning, Lin Huang, Zhongwei Yuan, Bihua Wu, Zehong Yan, Shoufen Dai, Bo Jiang, Youliang Zheng, Dengcai Liu

**Affiliations:** ^1^State Key Laboratory of Crop Gene Exploration and Utilization in Southwest China, Sichuan Agricultural University, Ya’an, China; ^2^Triticeae Research Institute, Sichuan Agricultural University, Ya’an, China

**Keywords:** alien introgression, marker, synthetic wheat, *Triticum aestivum*, Triticeae

## Abstract

Breeding progress in most crops has relied heavily on the exploitation of variation within the species’ primary gene pool, a process which is destined to fail once the supply of novel variants has been exhausted. Accessing a crop’s secondary gene pool, as represented by its wild relatives, has the potential to greatly expand the supply of usable genetic variation. The crop in which this approach has been most strongly championed is bread wheat (*Triticum aestivum*), a species which is particularly tolerant of the introduction of chromosomal segments of exotic origin thanks to the genetic buffering afforded by its polyploid status. While the process of introgression can be in itself cumbersome, a larger problem is that linkage drag and/or imperfect complementation frequently impose a yield and/or quality penalty, which explains the reluctance of breeders to introduce such materials into their breeding populations. Thanks to the development of novel strategies to induce introgression and of genomic tools to facilitate the selection of desirable genotypes, introgression breeding is returning as a mainstream activity, at least in wheat. Accessing variation present in progenitor species has even been able to drive genetic advance in grain yield. The current resurgence of interest in introgression breeding can be expected to result in an increased deployment of exotic genes in commercial wheat cultivars.

## Introduction

The process of crop domestication, in which selection is applied to a population of wild plants, inevitably introduces a major genetic bottleneck (Simmonds, 1976). After many centuries of farmer-based selection, the late 19th century ushered in the hugely successful era of scientific plant breeding, a process which is predicated on creating novel combinations of the genetic variants represented in the crop’s primary gene pool (Borlaug, 1983). While continuing to deliver genetic advances in many crops, the evidence now suggests that a yield plateau has been reached in certain crops, leading to concerns that the still growing global demand for plant-based products (food, feed, fiber, and industrial products such as starch and oil) will not be easily met without innovations being made in breeding technology (Tester and Langridge, 2010). First and foremost is the need therefore to expand the genetic base of the most intensely bred crop species, notably maize, rice, and wheat.

The secondary gene pool of a crop species is populated by its wild (as well as, in some cases, its cultivated) relatives. Accessing this gene pool typically requires *ab initio* the creation of a wide hybrid between the domesticate and one of its related species. Reducing the representation of the exotic parent’s genome is most straight-forwardly achieved by backcrossing the hybrid to the domesticate (Anderson and Hubricht, 1938; Anderson, 1949), resulting in so-called “introgression” materials. While creating a viable wide hybrid is generally considered to require human intervention, wide hybrids clearly occur in the wild, as witnessed by the evolution of many natural allopolyploids, such as tetraploid and hexaploid wheat, oat, cotton, sugarcane, canola, coffee, and tobacco (Ribeiro-Carvalho et al., 2004; Dvorak et al., 2006; Udall and Wendel, 2006; Soltis et al., 2009). Introgression has made some significant contributions to crop improvement: an example is the introduction into rice of a gene conditioning male sterility gene from its wild relative *Oryza rufipogon*, which has facilitated the production of plentiful and affordable F_1_ hybrid grain, and thereby led to the widespread exploitation of heterosis (Yuan, 1966). In another example, a chromosome comprising a segment of a wheat chromosome fused to one from rye (*Secale cereale*) by the early 1990s was present in almost one half of the bread wheat cultivars bred in 17 countries (Rabinovich, 1998). Polyploid species such as wheat tend to be better able than diploid ones to tolerate the presence of exotic chromatin due to the buffering provided by the presence of multiple homeologs (Sears, 1956; Dubcovsky and Dvorak, 2007).

Bread wheat represents a case model for introgression breeding. The species belongs to the Triticeae tribe which houses over 500 species, including several other important crop species [macaroni wheat (*T. durum*), barley (*Hordeum vulgare*), and rye] and fodder species (Wang et al., 1994; Yen et al., 2005). The genome of each members of the tribe comprises a combination of 26 related, but distinct genomes (Liu et al., 2017a). The first reported wide hybrids made between Triticeae species date back to an attempt to cross bread wheat with rye by Wilson (1876). The literature describing so-called “chromosome engineering” (Sears, 1972) is dominated by experiments conducted using bread wheat as the recipient. Wide hybrids involving Triticeae species have been used to understand a number of basic phenomena, such as the loss of centromeric histone H3 which accompanies chromosome elimination (Sanei et al., 2011), the formation of unreduced gametes underlying spontaneous chromosome doubling (Hao et al., 2014) and the restriction of chromosome pairing in allopolypoids to homologs (Sears, 1976).

Many papers have been published on the theme of introgression in wheat, and some useful reviews have been assembled by Sears (1981), Friebe et al. (1996), Kole (2011), and Mason (2017). The aim here was therefore not to provide another review of wheat introgression, but rather to discuss how some recently developed breeding strategies are contributing to a resurgence of interest in introgression breeding. Although the focus of the review is very much concentrated on wheat, many of these issues will be equally applicable to other crop species.

## The Bread Wheat Gene Pool

The gene pool concept proposed by Harlan and de Wet (1971) rests on a classification of the ease/difficulty of generating viable sexual hybrids, which is the *sine qua non* of introgression. In their scheme, a crop species’ near relatives fall into either the primary (GP-1), secondary (GP-2) or tertiary (GP-3) gene pools. Membership of the three gene pools in Triticeae is determined largely by the species’ genomic constitution (Jiang et al., 1993; Feuillet et al., 2008). In the case of common wheat as the recipient species, we here re-consider Triticeae species as one of four types based on the genome constitution and the ease/difficulty of introgression breeding.

*GP-1* species share the same three genomes (B, A, and D) which make up the bread wheat genome. These species, such as *T. spelta* and *T. macha*, are fully sexually compatible with bread wheat. As a result, introgression is achievable through conventional crossing and selection, since recombination between the chromosomes of the recipient bread wheat and the donor relative is effectively unrestricted. Some back-crossing is usually required to restore the bread wheat genetic background.

*GP-2* species share only some of the bread wheat genomes. Two prominent examples are *T. turgidum*, a group of tetraploid wheats of genomic constitution BA and *Aegilops tauschii* (goatgrass), the donor of bread wheat’s D genome. Like GP-1 species, these are generally readily crossable with wheat, although because of their unbalanced chromosome constitution, the resulting hybrids are typically only poorly fertile. Nevertheless, introgression is relatively straight-forward as it can be affected by homologous recombination. These species are of particular value as a genetic resource for bread wheat improvement. As a result, there has been a concerted, worldwide effort to mine variation from these GP-2 species (particularly *Ae. tauschii*) by creating synthetic hexaploid wheats (SHWs), which replicate the natural wide cross responsible for the speciation of hexaploid wheat (Mujeeb-Kazi et al., 1996).

*GP-3* species share no homologous genomes with bread wheat; they thus include the majority of the Triticeae species, including the two domesticates rye (R genome) and barley (H genome). For these species, not only are wide crosses less easy to develop (generally requiring embryo rescue because the hybrid endosperm fails to develop), but also introgression has to rely on inducing either homeologous recombination or a chromosome breakage-fusion event.

*GP-2/3* species feature at least one genome present in bread wheat, alongside at least one which is homeologous. Examples include *Ae. cylindrica* (CD) and *Ae*. *ventricosa* (DN) and the large number of synthetic wheat × rye amphiploids (BAR and BADR), referred to as triticales. Here, introgression is possible via homologous recombination, provided that the target gene resides within a chromosome belonging to the homologous genome. Otherwise, as for the GP-3 species, introgression has to rely on inducing either homeologous recombination or a chromosome breakage-fusion event.

In addition, it has been proposed to add a fourth gene pool (GP-4) to acknowledge the potential of transgenesis (Suslow et al., 2002; Kumar and Rustgi, 2014) and somatic hybridization (Xia et al., 2003) to introduce genes without any requirement for the prior formation of a sexual hybrid. While wheat’s GP-4 in principle harbors every living organism, from microbe to mammal, it also includes a number of related species, notably sorghum (*Sorghum bicolor*), Job’s tears (*Coix lacryma-jobi*), and Cogon grass (*Imperata cylindrica*) (Liu et al., 2014); while it is possible to culture *in vitro* immature hybrid embryos formed when wheat is pollinated by these species and to regenerate viable plants, the non-wheat chromosomes are rapidly eliminated during the zygote’s early cell divisions, so that the regenerants are effectively wheat haploids. Note in passing that access to transgenic crops is limited in many countries, meaning that GP-4 represents at best a theoretical resource, at least for the present.

## Barriers to Exploiting Introgression Materials in Wheat Improvement

While a substantial research investment has been made into creating introgression materials, their impact on wheat improvement has been relatively modest. The major reason for this outcome is that many of genotypes have proven to be defective in terms of plant type, grain yield and/or grain quality, reflecting a combination of linkage drag and an inadequate level of genetic complementation.

When a target gene is introduced, whether this is achieved using recombination or chromosome breakage/fusion, it will inevitably be accompanied by other genes linked to it on the introgressed segment; some of these genes may have deleterious consequences on the plant’s agronomy, productivity or grain quality. The ideal therefore is to engineer a transfer which involves as short an introgressed segment as possible. Linkage drag can potentially be overcome where the introgression has been achieved by homologous recombination, since the length of the segment can in principle simply be reduced by inducing further rounds of recombination enabled by a program of backcrossing. Linkage drag is more difficult to negate in materials which have been generated as a result of a homeologous recombination event, because no further recombination will occur once the wild type restriction over the pairing of homeologs has been restored (Sears, 1976).

The potential for inadequate genetic complementation becomes increasingly likely where the donor species is only distantly related to the recipient. In this case, the donor and recipient species have been isolated from one another over such a long period that they will have diverged substantially at the genetic level. As a result, the gene content of the introgressed exotic segment and the wheat segment which has been replaced may not be the same, leading to a progeny in which the introgressed genes cannot fully complement those present on the replaced wheat chromosome segment. This situation can lead to deleterious effects on the plant’s agronomic performance (Birchler and Veitia, 2012). While genetically unbalanced genotypes can be informative for the purpose of genetic analysis, they are seldom of value in the context of varietal improvement.

The genetic background in which an exotic transfer has been engineered can also discourage the breeder take-up of introgression materials. Much of the research effort in bread wheat has been focused on the Sichuan province landrace Chinese Spring (CS), because of its choice for the development of the aneuploid and mutant stocks required for chromosome engineering (Sears and Miller, 1985). This choice has had some unfortunate consequences, since the phenotype of CS is deficient with respect to several key quantitative traits, and has proven difficult to correct (Liu et al., 2018). Nevertheless, a substantial body of germplasm harboring introgression products has been developed by various programs, although so far their impact on wheat improvement has been minimal, given that breeders are unwilling to break up the constellation of favorable alleles which they so laboriously assembled through many years of crossing and selection. Some recent advances in introgression methodology and the development of genomic resources have the potential to overcome some of the problems associated with the exploitation of introgression materials.

## The Use of Double Monosomics to Generate Robertsonian Translocations

Unpaired meiotic chromosomes (univalents) have a tendency to spontaneously break at their centromere during anaphase I to form two fragments. This process can give rise to a Robertsonian translocation (RobT) where two different chromosomes simultaneously break in the same cell, since chromosomal fragments readily fuse with one another (Robertson, 1916; Sears, 1952). Balancing or compensating RobTs (cRobTs), in which a chromosome arm becomes fused to the opposite arm of its homeolog, represent a key intermediate in the process of inducing introgression in the situation where homologous recombination is not feasible. A small number of cRobTs have indeed had a considerable impact on wheat improvement; the most notable examples are the wheat–rye translocation 1BL.1RS, in which the short arm of wheat chromosome 1B has been replaced by the short arm of rye chromosome 1R (Mettin et al., 1973; Zeller, 1973), and the 6AL.6VS translocation, in which the long arm of wheat chromosome 6A has become fused to the short arm of *Dasypyrum villosum* chromosome 6V. The former cRobT was ubiquitous among high yielding CIMMYT and European wheats during the closing years of the 20th century, while the latter one, which carries a gene determining resistance to the foliar pathogen *Blumeria graminis* (powdery mildew), is present in many current Chinese cultivars (Cao et al., 2011).

**FIGURE 1:**
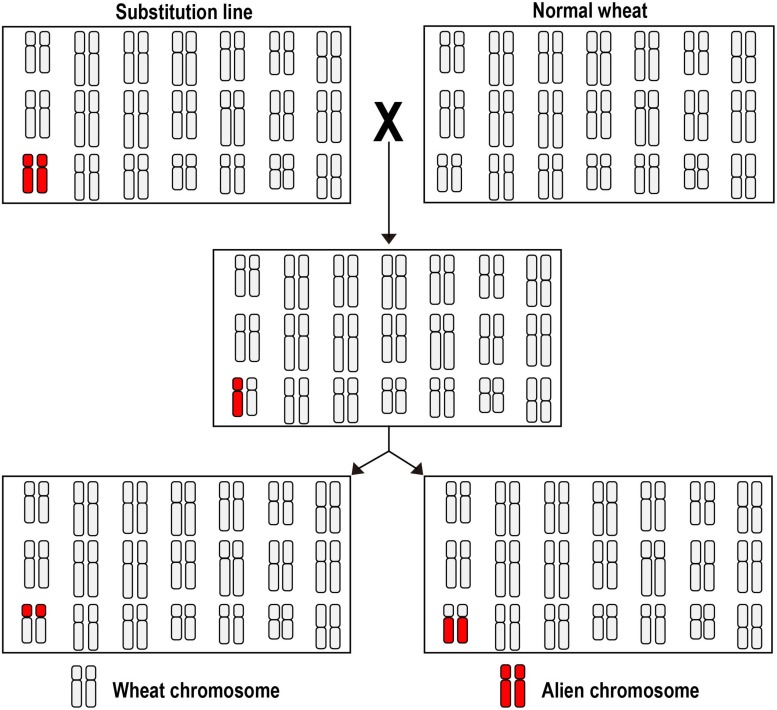
The double monosomic method use to induce cRobTs. A euploid cultivar is crossed with a whole chromosome substitution line in which an exotic chromosome has replaced its wheat homeolog. The resulting F_1_ hybrid carries two chromosomes which remain as univalents at meiosis metaphase I, giving the opportunity for a joint breakage/fusion event at anaphase I to create a cRobT.

Constructing a wheat plant in which two chromosomes are present as monosomics is a relatively straight-forward procedure given the range of cytogenetic stocks which have been assembled (Davies et al., 1985; Lukaszewski, 1993, 1997; Friebe et al., 2005). A list of some successful inductions is presented in Table 1. The principle of the double monosomic method is based on the certainty that, thanks to the suppression of homeolog pairing, the two monosomes will remain unpaired at meiotic metaphase I (giving rise to a meiotic constitution of 20′′ + 2′); selfed progeny of such a plant are subsequently screened, using a combination of genetic markers and karyotyping, to detect *de novo* cRobT products. Both marker and karyotyping technology have benefited from progress in DNA analysis, the former taking the form of DNA-based markers such as microsatellites and single nucleotide polymorphisms (SNPs), and the latter exploiting either genomic or fluorescence *in situ* hybridization (GISH and FISH). Double monosomics are most easily generated by crossing a whole chromosome substitution with a euploid plant (Figure 1): for instance, Ardalani et al. (2016) crossed a 6Eb(6D) substitution with the cultivar “Roushan,” and among 80 F_2_ segregants of the resulting 20′′ + 6D′ + 6Eb′ plant, it was possible to identify, using a PCR-based screen, a plant carrying a 6DL.6EbS cRobT. Similarly, Hao et al. (2018) only needed to screen 69 segregants to uncover a 6AL.6RS cRobT. A second approach begins with a cross between an established monosomic line (20′′ + 1′) and an addition line (21′′ + 1′′), as exemplified by Danilova et al. (2018); in this case, each of the three stocks monosomic for a group 7 homeolog (20′′ + 7A′, 20′′ + 7B′ and 20′′ + 7D′) were crossed with a line carrying a disomic dose of barley chromosome 7H (21′′ + 7H′′); selection among the resulting F_1_s was then made for plants having a somatic chromosome number of 42, which were expected to be of meiotic constitution 20′′ + 7A/B/D′ + 7H′. A marker-based screen of the progeny of these selections was successful in identifying all six potential cRobTs involving each arm of the barley chromosome and its opposite wheat arm, and these were confirmed using GISH analysis.

**TABLE 1:** Examples of compensating Robertsonian translocations generated by double monosomic method.

**Donor species**	**Target traits and genes**	**Translocation chromosome**	**References**
*Haynaldia villosa*	Resistance to wheat spindle streak mosaic virus	4VS⋅4DL	Zhang Q. et al., 2005*
*H. villosa*	Increasing gluten strength	1V#3S⋅1DL	Zhao et al., 2010**
*H. villosa*	Stem rust *Sr52*	T6AS⋅6V#3L	Qi et al., 2011**
*H. villosa*	/	A set of RobTs involving 12 *D. villosum* chromosomes	Liu et al., 2011a**
*Aegilops searsii*	Stem rust resistance	3S^*s*^S⋅3AL, 3S^*s*^S⋅3BL, 3S^*s*^S⋅3DL	Liu et al., 2011b*
*Thinopyrum intermedium*	Resistance to streak mosaic virus	7BS⋅7S#3L	Liu et al., 2011c**
*Th. intermedium*	Stem rust *Sr44*	7J#1S⋅7DL	Liu et al., 2013**
*Th. bessarabicum*	High Fe and Zn contents	6E^*b*^S⋅6DL	Ardalani et al., 2016*
*Th. elongatum*	Improving flour quality	1AS.1EL	Tanaka et al., 2017*
Rye	Stem rust *Sr59*	2DS⋅2RL	Rahmatov et al., 2016*
Rye	Powdery mildew *Pm56*	6RS⋅6AL	Hao et al., 2018*
Barley	β-glucan CslF6; long spikes	A complete set of six RobTs	Danilova et al., 2018**
Barley	Salt tolerance and β-glucan content	7BS⋅7HL	Türkösi et al., 2018**

*The production of double monosomic plants by crossing between substitution and normal lines; **The production of double monosomic plants by crossing between monosomic and addition lines.

## Exploiting the *Ph* System to Generate Sub-Chromosomal ARM Introgressions

While Sears (1956) showed that it was possible to transfer a segment of an *Ae. umbellulata* chromosome carrying a gene conditioning leaf rust resistance into wheat by irradiation with X-rays, chromosome engineering was greatly accelerated following the discovery that the suppression of meiotic pairing between wheat homeologs is under relatively simple genetic control. The most important locus is *Ph1*, mapping to *c*hromosome arm 5BL (Okamoto, 1957; Riley and Chapman, 1958), and now known to have formed as a result of the insertion of a segment of subtelomeric heterochromatin within a cluster of *cdc2*-related genes (Griffiths et al., 2006). Deleting the *Ph2* locus (mapping to chromosome arm 3DS) has an intermediate effect on pairing suppression (Mello-Sampayo, 1971; Sutton et al., 2003), while a large effect locus has recently been mapped to chromosome arm 3AL by Fan et al. (2019). A number of minor effect loci were identified by Sears (1976). Importantly for chromosome engineering technology, the suppression of homoelog pairing imposed by both *Ph1* and *Ph2* extends beyond wheat itself, and thus can be exploited to induce recombination between a number of wheat chromosomes and their homelogs from related species (Riley et al., 1968; Koebner and Shepherd, 1985, 1986, 1987; Ceoloni et al., 1992).

**FIGURE 2:**
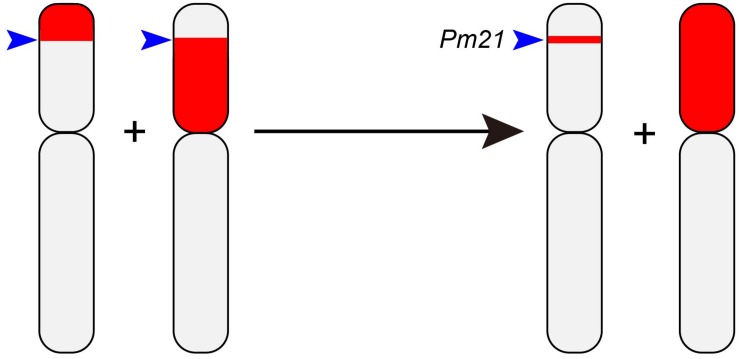
The introgression of a small fragment of *Ha. villosa* chromosome 6V into a distal site of wheat chromosome arm 6AS. Two independent introgression lines were inter-crossed: one carries the terminal portion of chromosome arm 6VS and the other a proximal segment, while both carry *Pm21*, which lies in the short common interstitial segment. As a result of a homologous recombination event in the F_1_ hybrid, segregants are generated which carry only the short interstitial segment [Figure redrawn from Lukaszewski and Cowger (2017)].

Since large translocations are prone to be deleterious because of linkage drag and/or inadequate genetic complementation, reducing their size, while maintaining the presence of the gene(s) targeted for introgression, is a desirable goal (Moore et al., 1993). Given that the success rate in inducing introgressions is quite low, and that conventional cytological methods are not capable of distinguishing between large and small introgression segments, pioneering attempts to achieve this goal were largely unsuccessful. Recent advances with respect to both chromosome manipulation and particularly in genetic marker technology, however, are changing this picture (Winfield et al., 2016; King et al., 2017, 2019; Koo et al., 2017). A list of successful examples is presented in Table 2. In a recent case example, an attempt to shorten the 6AL.6VS translocation has been described by Lukaszewski and Cowger (2017). The target gene on the *Ha. villosa* chromosome arm is *Pm21*, which protects against infection by *B. graminis* (Cao et al., 2011). To reduce the length of 6VS chromatin present, the cRobT was introduced into a background deficient for *Ph1* to generate plants of constitution 19′′ + 5B′′ (*ph1b*) + 6AL.6VS′ + 6A′. A marker-based screen of 997 progeny allowed for the identification of 29 new translocations involving combinations of segments from chromosome arms 6AS and 6VS. The critical step was then to cross a plant containing a translocation comprising the terminal segment of 6VS (including *Pm21*) with one (also including *Pm21*) which carried a translocation comprising the proximal end of 6VS; since the two translocations shared a small fragment 6VS, it was then possible to exploit homologous recombination to generate a chromosome harboring a small interstitial segment of 6VS (including *Pm21*) inserted into the sub-terminal region of 6AS (Figure 2).

**TABLE 2:** Examples of small translocations generated by manipulation with *ph1b*.

**Alien donor species**	**Target traits and genes**	**Translocation chromosomes**	**References**
*Aegilops umbellulata*	*/*	1U/1B; 1U/1D	Koebner and Shepherd, 1987
*Aegilops speltoides*	Leaf rust *Lr47*; green bug *Gb5*	7S/7A	Dubcovsky et al., 1998
*Ae. speltoides*	Stem rust/*Sr39*	2S/2B	Niu et al., 2011
*Ae. speltoides*	/	2S/2B	Zhang et al., 2017, 2018
*Ae. speltoides*	Tan spot *TsrAes1*; *Septoria nodorum* blotch *SnbAes1*	2S/2B	Zhang et al., 2019b
*Aegilops sharonensis*	*Lr56*/*Yr38*	T6AS⋅6AL-6S^*sh*^/6A	Marais et al., 2010
*Aegilops searsii*	Powdery mildew *Pm57*	2S^*s*^#1/2B	Liu et al., 2017b
*Secale cereale*	Removing *secalin* of T1RS.1BL	T1RS⋅1BL/1B; T1RS⋅1BL/1D	Koebner and Shepherd, 1985, 1986
*S. cereale*	Removing *secalin* of T1RS.1BL	T1RS⋅1BL/1B	Lukaszewski, 2000
*S. cereale*	/	T2RS⋅2BL/2B; T2BS⋅2RL/2B	Lukaszewski et al., 2004
*S. cereale*	Removing *secalin* of T1RS.1BL	T1DS-1RS-1DS⋅1DL/1D	Anugrahwati, 2009
*Thinopyrum ponticum*	Leaf rust *Lr19*; yellow pigment	T7DS⋅7DL-7EL/7A; T7DS⋅7DL-7EL/7D	Zhang W. et al., 2005
*Th. ponticum Th. intermedium*	Barley yellow dwarf virus *Bdv2*; Rust resistance *Lr19* and *Sr25*	T7DS⋅7J?/T7DS⋅7DL-7S?	Ayala-Navarrete et al., 2007
*Th. intermedium*	Wheat streak mosaic virus *Wsm3*	T7BS⋅7S#3L/7B	Danilova et al., 2017
*Th. elongatum*	/	2E/2B	Zhang et al., 2017, 2018
*Hordeum vulgare*	/	4H^*ch*^/4D	Rey et al., 2015
*H. vulgare*	β*-*glucan synthesis *HvCslF6*	T7HL⋅7AS/7A; T7HL⋅7BS/7B; T7HL⋅7DS/7D	Danilova et al., 2019
*Elymus tsukushiensis*	*Fusarium* head blight *Fhb6*	1Ets#1S/1A	Cainong et al., 2015
*Haynaldia villosa*	*Powdery mildew Pm21*	T6VS⋅6AL/6A	Lukaszewski and Cowger, 2017
*Ha. villosa*	Wheat yellow mosaic virus *Wss1*	T4VS⋅4DL/4D	Zhao et al., 2013; Dai et al., 2019
*Ha. villosa*	Stem rust *Sr52*	T6AS-6V#3L/6A	Li et al., 2019

## Homology-Directed Introgression Aimed at Enhancing Complex Traits

Most of the introgression experiments reported in the literature have sought to transfer a single gene trait, most prominently resistance to disease. Most breeders’ traits – notably grain yield – are, however, under polygenic control. The “omnigenic model” has been developed as a means of accounting for the observation that despite the inheritance of certain human diseases being due to the effect of many genomically dispersed genes, some of these genes encode products having no obvious connection to the expression of the disease (Boyle et al., 2017). The model implies that gene regulatory networks are sufficiently interconnected with one another such that all genes expressed in disease-relevant cells are liable to affect the functions of core disease-related genes and that most of the heritability can be explained by effects on genes outside core pathways. An equivalent situation probably exists with respect to the genetic determination of yield, which is clearly complex given the large number of quantitative trait loci which have been identified as contributing to the trait. A consequence of the hexaploid status of bread wheat is that interactions between homeologous genes are likely an important component of the overall genetic determination of yield-related traits (Flood et al., 1992; Yang et al., 2017). A further consequence is that the triplication of many genes in bread wheat, arising from the presence of three homeologs, provides a buffering effect, such that allelic variation at one of the copies may result in just a minor phenotypic effect (Dubcovsky and Dvorak, 2007). Thus, although variation at a single locus may well have an insubstantial effect on grain yield, combining a number of novel introgression segments into a single genotype has the potential to generate a significant positive effect. This possibility has been validated by the experience with SHWs, pursued originally by CIMMYT in the last decade of the 20th century, and later much expanded, both at CIMMYT and elsewhere. It has become clear that introgressing multiple segments from an SHW parent can significantly enhance the yield potential of wheat (Hoisington et al., 1999; Coghlan, 2006; Warburton et al., 2006; Dreisigacker et al., 2008; Trethowan and Mujeeb-Kazi, 2008; Yang et al., 2009; Li et al., 2018; Hao et al., 2019; Zhang et al., 2019a). The Chinese cultivar Chuanmai 42, for example, which has as one of its parents the CIMMYT SHW Syn769 (*T. durum* cv. Decoy 1/*Ae. tauschii* 188), recorded a yield of more than 6 t/ha in Sichuan regional trials, out-performing the commercial check variety by 20% over two consecutive years (Yang et al., 2009). It has been estimated that farmers using this cultivar would gain an extra 0.5–0.8 t/ha of grain (Li et al., 2014b). The recently released cultivars Shumai 580, Shumai 969, and Shumai 830 (Hao et al., 2019) were all bred from an SHW derived from the cross *T. turgidum* AS2255/*Ae. tauschii* AS60. Shumai 580 out-performed the best local check cultivar by 56% (equivalent to ∼2 t/ha) in a yield trial in Yunnan province, while Shumai 969 has remained the highest yielding cultivar in Sichuan trials over the past decade. The yield per spike achieved in Sichuan province by Shumai 830 has been consistently higher than any other commercial cultivar’s.

## Sequence-Based Analysis of the Genomes of SHW Derived Cultivars

A characterization of the introgressions carried by the three Shumai cultivars, as assessed at the sequence level by Hao et al. (2019), has shown that the ratio of the SHW parent’s DNA retained lay in the range 12.4–15.0%. This outcome coincides well with the predicted ratio of 12.5% based on the assumption that the breeding strategy employed did not discriminate against SHW alleles (except for those responsible for visible deleterious traits such as shattering, lateness and tallness). The genomes of the three Shumai cultivars featured a unique set of introgressions from SHW-L1: alleles from the B genome of the SHW genome were prominent in Shumai 580, those from the D genome in Shumai 969 and those from the A genome in Shumai 830. Although SHWs themselves tend to be poor agronomic performers, an analysis of a recombinant inbred population formed from a cross between SHW-L1 and an elite cultivar has shown that nearly a half (40/86) of the positive alleles at loci affecting yield were inherited from the SHW parent (Hao et al., 2019). Similar phenomenon has also been observed in other plant systems such as tomato (de Vicente and Tanksley, 1993). In addition to these quantitative trait loci, the Shumai cultivars inherited SHW alleles at *Ppd-A1*, *Vrn-A1*, *Vrn-B1*, *TaTEF*, *GPC*-*2*, *TaGASR7*, *TaGW2*, *TaCKX6*, and *TaSus1*, all of which are genes having a known impact on productivity (Nadolska-Orczyk et al., 2017). A similar representation of SHW alleles in materials selected from advanced backcross populations has been reported by Huang et al. (2003, 2004). The take home message is that while SHWs in themselves cannot compete with elite cultivars, they do represent a valuable source of beneficial alleles, so there is every reason to include them in bread wheat improvement programs.

In addition, SHW is different from bread wheat since its newly synthetic process may introduce transcriptome shock (Li et al., 2014a, 2015). Some marked differences have been noted between the transcriptomes of SHWs and conventional cultivars (Hao et al., 2017; Ramírez-González et al., 2018), resulting in the proposition that aspects of regulatory control differ (Li et al., 2015). Thus, besides the clear contribution of DNA sequence polymorphism, SHWs probably also deliver variation at the RNA level.

## A Strategy for Breeding Based on SHWs

The successful use of unadapted germplasm requires the introduction of genes determining the target trait(s) and selection against those determining deleterious traits. It has been conventionally held for a long time that using unadapted germplasm as a parent in a breeding program is inherently risky, since the size of breeding population required to breed these deleterious traits out may be uneconomically large. The indications are, however, that the efficiency of introgression breeding can be improved by adopting more focused strategies for both crossing and selection. The advanced backcross quantitative trait locus approach proposed by Tanksley and Nelson (1996) provides a method to retain favorable alleles inherited from the exotic donor parent while returning the background to that of the elite recipient. The concept has been applied to introgression programs in tomato (Tanksley et al., 1996), rice (Xiao et al., 1998), and bread wheat (Huang et al., 2003, 2004; Liu et al., 2006). However, in commercial practice, it has become evident that the necessity for extensive backcrossing has been exaggerated. Of 46 SHW-based cultivars released in 15 countries in the period 2003–2017, only four have been based on backcrossing to an elite cultivar (Li et al., 2018). Rather, the experience has been that top-crossing is a more effective approach, perhaps because of the unlikelihood that any single elite cultivar has the genetic content to neutralize the many defects present in an SHW, whereas including two or more elite parents increases the chance of success.

**FIGURE 3:**
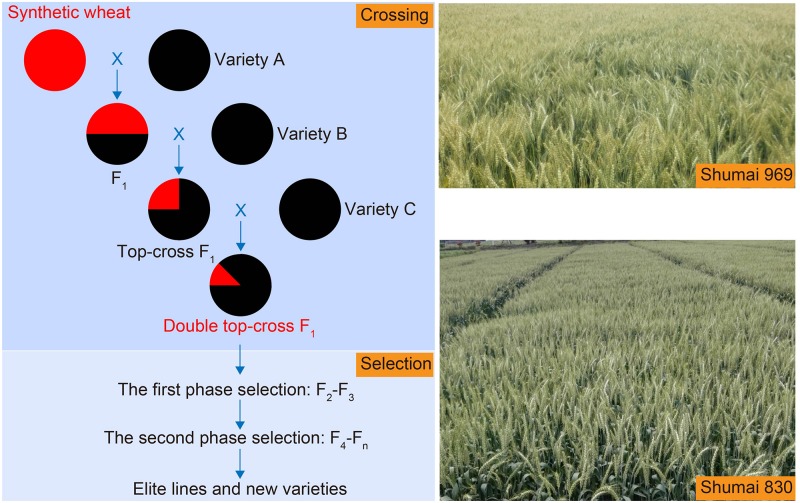
The double top-cross (DTC) and two phase selection (2PS) strategies deployed to manage introgression into elite germplasm from an SHW. The DTC populations retain on average 12.5% of the nuclear genome of the SHW parent. The aim of the first selection phase, carried out in the F_2_ and F_3_ generations, is to eliminate serious agronomic defects, while that of the second phase (applied from the F_4_ generation onward) is to improve yield. Shumai 969 and Shumai 830 were both bred using this strategy.

In the authors’ hands, an effective strategy has proven to be one based on a double top cross (DTC), followed by two phases of selection (2PS) (Hao et al., 2019; Figure 3). The scheme, summarized as SHW-L1/B//C///D (where B through D indicate three independent elite cultivars), implies that at its end the population’s predicted mean proportion of nuclear DNA inherited from SHW-L1 will be 12.5%. During the F_2_ and F_3_ generations, the population size was reduced by selecting against tough glumes, late maturity, tall stature and yellow rust susceptibility, while selection from the F_4_ onward was directed at yield. The program has already led to the release of three cultivars: Shumai 580 and Shumai 969 both emerged from rather small populations (∼100–200 F_2_ and ∼100–200 F_3_ plants), whereas the selection of Shumai 830 required a more conventional population size, because one of its parental lines was a less well established cultivar. A fourth release (Shumai 114) is imminent. Overall, the indication is that the DTC-2PS strategy can be highly effective for introgressing material from an SHW.

The effectiveness of the DTC-2PS strategy relies on a number of factors. Firstly, it assumes that the SHW donor harbors plentiful genetic variation, allowing introgression to potentially contribute to the improvement of extant traits or even the creation of novel ones. It has been suggested that inheritance of the rapid grain filling trait exhibited by SHW-L1 partially underlies the improved yield potential of the three Shumai cultivars (Hao et al., 2019). Secondly, the DTC scheme combines allelic variation from more than one elite cultivar, providing a wider pool of alleles for generating novel combinations: each of the elite cultivars has its specific constellation of favorable alleles available to interact with the donor’s genome. Thirdly, the early generations are used to select for major genes controlling the important domestication traits, leaving behind a much smaller population for the breeder to handle the issue of yield advance.

As demonstrated by Li et al. (2020), the DTC-2PS strategy has also been successfully deployed to improve the genetic background of a line harboring a 6AL.6RS wheat/rye translocation, valued for the presence of *Pm56* (Hao et al., 2018). Two genetic backgrounds provided the starting material: one was CS and the other the Sichuan province landrace Kaixian-luohanmai. The strategy is quite general, so could readily be used to widen the genetic base of any crop species. It is of particular relevance to allopolyploid species where the progenitor parents are known (for example in cotton, 4x *Brassica* spp. and groundnut), since the domesticate has become genetically isolated from the wild progenitor species and its genetic base has been narrowed as a result of domestication and subsequent breeding (Stebbins, 1950; Buckler et al., 2001). The expectation is that introgression from exotic germplasm will feature strongly in future crop improvement programs.

## Author Contributions

MH and DL contributed to the conception and design of the study and wrote the first draft of the manuscript. All authors contributed to the manuscript revision, read, and approved the submitted version.

## Conflict of Interest

The authors declare that the research was conducted in the absence of any commercial or financial relationships that could be construed as a potential conflict of interest.
